# Cartilage defect location and stiffness predispose the tibiofemoral joint to aberrant loading conditions during stance phase of gait

**DOI:** 10.1371/journal.pone.0205842

**Published:** 2018-10-16

**Authors:** Lianne Zevenbergen, Colin R. Smith, Sam Van Rossom, Darryl G. Thelen, Nele Famaey, Jos Vander Sloten, Ilse Jonkers

**Affiliations:** 1 Department of Movement Sciences, Human Movement Biomechanics Research Group, KU Leuven, Leuven, Belgium; 2 Institute for Biomechanics, ETH Zürich, Zürich, Switzerland; 3 Department of Mechanical Engineering, University of Wisconsin-Madison, Madison, WI, United States of America; 4 Department of Biomedical Engineering, University of Wisconsin-Madison, Madison, WI, United States of America; 5 Department of Orthopedics and Rehabilitation, University of Wisconsin-Madison, Madison, WI, United States of America; 6 Department of Mechanical Engineering, Biomechanics Section, KU Leuven, Leuven, Belgium; University of California Berkeley, UNITED STATES

## Abstract

**Objectives:**

The current study quantified the influence of cartilage defect location on the tibiofemoral load distribution during gait. Furthermore, changes in local mechanical stiffness representative for matrix damage or bone ingrowth were investigated. This may provide insights in the mechanical factors contributing to cartilage degeneration in the presence of an articular cartilage defect.

**Methods:**

The load distribution following cartilage defects was calculated using a musculoskeletal model that included tibiofemoral and patellofemoral joints with 6 degrees-of-freedom. Circular cartilage defects of 100 mm^2^ were created at different locations in the tibiofemoral contact geometry. By assigning different mechanical properties to these defect locations, softening and hardening of the tissue were evaluated.

**Results:**

Results indicate that cartilage defects located at the load-bearing area only affect the load distribution of the involved compartment. Cartilage defects in the central part of the tibia plateau and anterior-central part of the medial femoral condyle present the largest influence on load distribution. Softening at the defect location results in overloading, i.e., increased contact pressure and compressive strains, of the surrounding tissue. In contrast, inside the defect, the contact pressure decreases and the compressive strain increases. Hardening at the defect location presents the opposite results in load distribution compared to softening. Sensitivity analysis reveals that the surrounding contact pressure, contact force and compressive strain alter significantly when the elastic modulus is below 7 MPa or above 18 MPa.

**Conclusion:**

Alterations in local mechanical behavior within the high load bearing area resulted in aberrant loading conditions, thereby potentially affecting the homeostatic balance not only at the defect but also at the tissue surrounding and opposing the defect. Especially, cartilage softening predisposes the tissue to loads that may contribute to accelerated risk of cartilage degeneration and the initiation or progression towards osteoarthritis of the whole compartment.

## Introduction

Articular cartilage defects of the knee are commonly diagnosed in healthy adults by magnetic resonance imaging (MRI) [1] and arthroscopy [2–4]. The highest incidence of articular cartilage defects is found in the medial femoral condyle [2–6]. The natural history of cartilage defects remains unclear, although they have been shown to often occur following trauma [2,7], but also repetitive subthreshold shear and torsional loads to the articular surface can result in injuries to the joint surface [8,9]. More specifically, articular cartilage defects occur most frequently in the load bearing area of the tibiofemoral joint when the knee is in extension [6]. Not surprisingly, during arthroscopy the prevalence of full-thickness articular cartilage defects without any concominant injuries is 36% in an athlethic population [10], whereas it is about 18% in the general population [2,5].

Cartilage defects may eventually cause pain and functional impairment [11] and predispose the patient to progressive degenerative changes leading to osteoarthritis (OA) [12,13]. Despite the evolution to degenerative arthritis being multifactorial, changes in mechanical properties caused by cartilage damage will affect joint homeostasis by altering the contact mechanics and mechanobiological response of tissue adjacent to the defect on the long term [14–16]. Furthermore, elevated contact stresses in the tibiofemoral joint have been associated with biological maladaptive responses and consequently the initiation of OA [17,18].

Due to the limited repair capacity of articular cartilage, the majority of isolated cartilage defects in the knee joint progresses to OA within 2 years when left untreated [19,20]. This holds not only for osteochondral defects but also for partial thickness defects [21]. Current therapeutic treatment of cartilage defects aims to alleviate the pathology-related symptoms, prevent progression of cartilage damage, and restore the mechanical function [22]. However, surgical treatments, such as microfracture, osteochondral allograft transplantation (OAT), autologous chondrocyte implantation (ACI), and resurfacing implants have varied success rates when evaluating clinical, histological and radiological outcomes and failure rates [23–25]. The main concerns with those treatment techniques and untreated healing of full thickness defects are the formation of fibrocartilage and structural changes of the subchondral bone [26,27]. Fibrocartilage, characterized by a large fraction of collagen I fibers next to the collagen II and chondrocytes typically present in hyaline cartilage, has inferior mechanical and biochemical properties compared to the latter [28,29]. Structural changes of the subchondral bone could result in advancement of the subchondral bone plate towards the joint surface, formation of osteophytes and subchondral bone cysts [26,30]. Both factors contribute to altering the mechanical stiffness of the articular surface, which may negatively affect the homeostasis of surrounding and opposing tissues in the joint [11].

Although important, few studies have investigated the treatment outcome as function of defect location [31]. Research has shown that clinical outcome of articular cartilage defects located in the central part of the medial compartment was significantly worse than the outcome of defects in other regions of the weight-bearing parts of the tibiofemoral joint when left untreated or following microfracture [13,32]. Moreover, microfracture treatment of defects located at the lateral femoral condyle resulted in the best clinical outcomes [33]. Conversely, one study found that the clinical outcome was better for medial than lateral defects for microfracture and ACI treatments [34] and that microfracture had the best results in the femoral condyle compared to the tibia plateau [35,36].

In order to better understand the impact of local cartilage defects on contact mechanics, their influence has been estimated experimentally *in vitro* using pressure sensitive films [14,37–39], or computationally using whole joint finite element models [40–44]. However, most of these studies focus only on femoral defects and are restricted to the evaluation of one static pose that may not be representative for the joint positions during common functional activities such as walking. One recent finite element study, showed that focal defects located at the central regions of the medial tibia were more vulnerable to loading compared to defects covered by the meniscus during gait [44]. Whereas another finite element study found that adjustment of the articular cartilage stiffness in the lateral tibia perturbed the mechanical response of the articular cartilage surrounding and opposing the lesion during static compression [43].

The aim of this study is to identify the influence of defect location in tibia and femur cartilage and defect stiffness on the magnitude and distribution of tibiofemoral cartilage contact pressure during human gait. We hypothesize that the defects located in the load bearing area of the tibiofemoral joint will cause a redistribution of the contact pressure to adjacent cartilage within the weight-bearing area of the articular surface. Increased stiffness caused by changes in the subchondral bone plate is expected to decrease the loading of the adjacent cartilage while increasing the pressure inside the defect. In contrast, decreased stiffness as present with cartilage softening due to matrix breakdown or the formation of fibrocartilage is expected to decrease the average pressure inside the defect. By documenting the complex loading environment and contact pressure distribution on the tibiofemoral cartilage surfaces in the presence of an articular defect, this study may provide insights in the understanding of mechanical factors contributing to cartilage degeneration and may assist in the definition of knee rehabilitation protocols in patients with cartilage defects in an otherwise healthy knee joint.

## Methods

### Experimental procedure

External loads and kinematic data were collected in 10 healthy subjects (5 males and 5 females, age: 30±8 years, weight: 64.63±4.08 kg, height: 1.75±0.06 m) during barefoot overground walking at their self-selected speed (1.35±0.15 m/s). Retro-reflective skin mounted markers were placed according to the plug-in-gait full body marker set, expanded with additional cluster markers on the thigh and shank and anatomical markers on the sacrum, medial femur epicondyles and medial malleoli [45]. The inclusion of cluster makers allowed for better tracking of the segments, whereas the anatomical markers improved model scaling [46]. Three-dimensional marker trajectories were captured with a 10-camera motion capture system (Vicon, Oxford Metrics, Oxford, UK) at 100 Hz. Simultaneously, ground reaction forces were measured using three force plates (AMTI, Watertown, MA, USA) embedded in the 10 m walkway at 1000 Hz. Marker trajectories and ground reaction forces were low-pass filtered with cut-off frequency of 6 Hz and 30 Hz, respectively. Three representative gait trials with valid force plate contact were selected for each subject for further processing. All procedures were approved by the ethics committee of the university hospital Leuven (s56093), and subjects gave written informed consent prior to data collection.

### Musculoskeletal model

Muscle and joint contact forces were computed using a generic musculoskeletal model of the lower extremity [47] including a customized knee joint definition that was previously validated[48]. This knee model included 6 degrees-of-freedom (DOF) tibiofemoral and patellofemoral joints. The passive restraints of the knee joint are provided by the major knee ligaments and joint capsule, represented by 14 bundles of non-linear springs [49]. Tibiofemoral and patellofemoral cartilage geometry were segmented from MRI images (Mimics Innovation Suite, Materialise, Belgium) from a young adult female (23 years, 1.65 m, 61 kg) [48]. The cartilage contact pressures (p) acting between articulating surfaces were computed using an extended elastic foundation model [50,51];

$$p=-\frac{(1-v_{1})E_{1}}{\left(1+v_{1})(1-2v_{1}\right)}\ln[1-\frac{d_{1}}{h_{1}}]=-\frac{(1-v_{2})E_{2}}{\left(1+v_{2})(1-2v_{2}\right)}\ln\left[1-\frac{d_{2}}{h_{2}}\right],$$ (1)

with,

$$d_{1}+d_{2}=d,$$ (2)

where p represents the contact pressure, and d_1_ and d_2_ represent the deflection of contacting cartilage surfaces. The system of equations (Eqs 1 and 2) is solved for each pair of contacting triangles (subscripts) in the cartilage meshes [52] given the elastic modulus (E), Poisson’s ratio (ν), and thickness (h) of each cartilage geometry. Surfaces were represented by uniform triangulated meshes with a maximum edge length of 2mm and uniform cartilage thickness of 2mm [53,54]. Biomechanical changes following cartilage injury were represented by a change in elastic modulus for selected triangles. A detailed description of this procedure is provided in the defect location section below. The full lower-extremity model was implemented in SIMM [55] with the Dynamics Pipeline (Musculographics Inc., Santa Rosa, CA) and SD/Fast (Parametric Technology Corp., Needham, MA) describing the multi-body equations of motion.

### Simulation of knee mechanics during gait

For each subject, the musculoskeletal model, including the tibiofemoral contact geometries, was scaled to match the subject-specific anthropometry and mass as determined in an upright static calibration trial. The joint angles that best agreed with the experimental marker positions during gait were computed by global optimization based inverse kinematics [56]. Within this step, secondary tibiofemoral (rotation, adduction and tibial translations) and patellofemoral degrees of freedom were a constrained function of knee flexion [47,57]. Next, the concurrent optimization of muscle activations and kinematics (COMAK) method was used to estimate the ligament forces, muscle forces, and tibiofemoral and patellofemoral contact pressure distribution at each frame of the gait cycle. During this simulation step only knee flexion was tracked while the secondary tibiofemoral and all patellofemoral DOF evolved as a result of cartilage contact, ligament and muscle forces [52]. Muscle redundancy was resolved by minimizing the weighted sum of muscle activations squared and the net potential energy due to contact while satisfying overall dynamic constraints [52,57].

### Defect location

**Fig 1:**
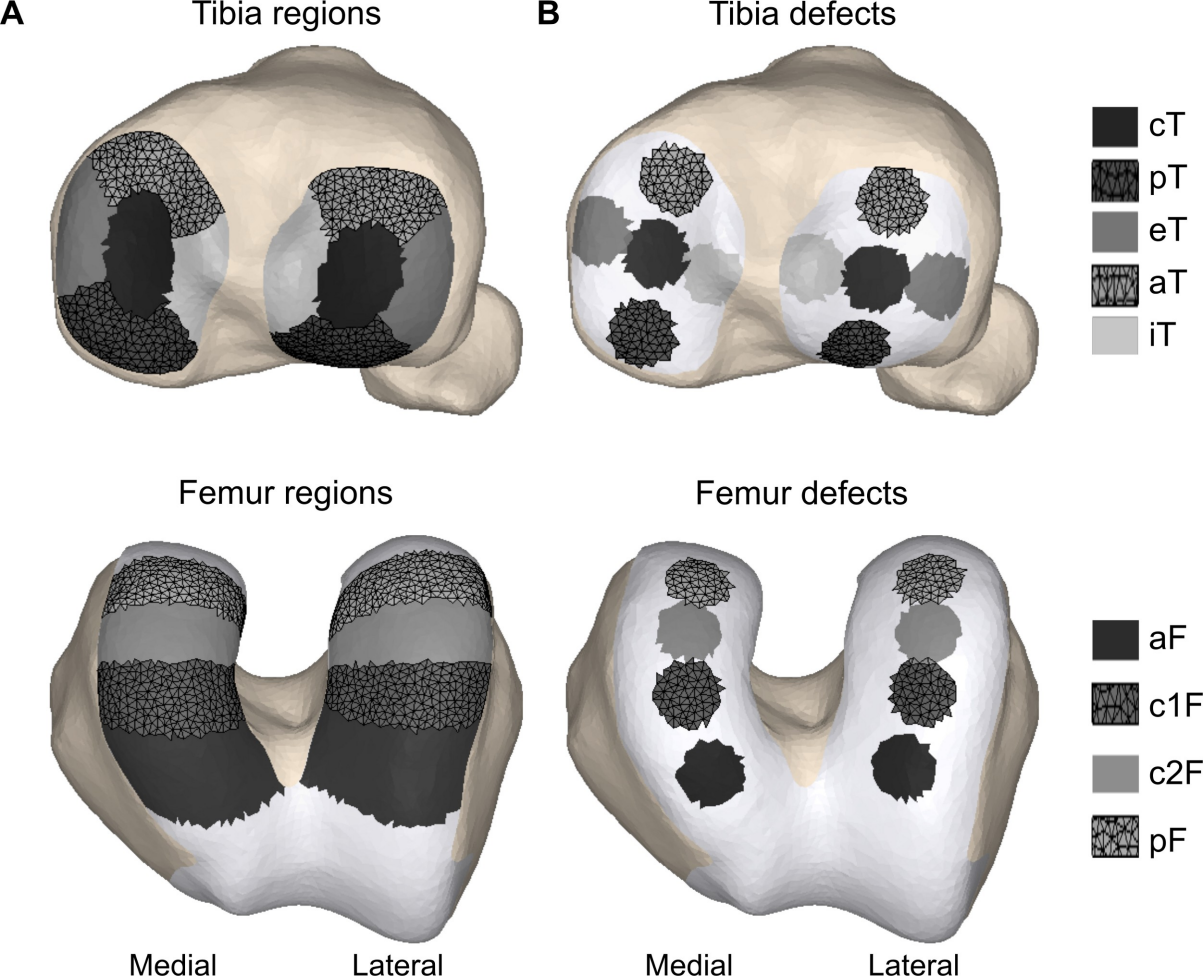
Model adaptations to simulate different cartilage defect locations in the tibia and femur articular surface. (A) To define the cartilage defect regions the tibia and femur articular surface were divided in 10 and 8 sub-regions, respectively. The medial and lateral tibia cartilage surfaces were each divided into five regions; cT—central, iT—internal, eT—external, aT—anterior and pT—posterior [58]. An ellipsoid defining the central tibia region covers 20% of the total cartilage area. The femur cartilage was first divided in medial and lateral compartment by a plane through the intercondylar notch perpendicular to the axis of the cylinder fitted onto the cartilage surface. Around the cylindrical axis, four angular segments of 30 degrees in anterior-posterior direction relative to the intercondylar notch were defined (aF—anterior, c1F - anterior-central, c2F - posterior-central and pF—posterior). (B) Circular defects of 100 mm^2^ were defined in the center of the defined sub-regions of the tibia and femur.

To evaluate the influence of altered mechanical properties at the defect site, we varied the elastic modulus of selected regions of interest, while keeping the properties of the surrounding cartilage unchanged. First, the medial and lateral tibia cartilage surfaces were each divided into five regions; central, internal, external, anterior and posterior [58], Fig 1. The medial and lateral femoral cartilage were each divided into four equal regions based on angular segments of 30 degrees in anterior-posterior direction relative to the intercondylar notch around the axis of a cylinder fitted onto the cartilage surface [59], Fig 1A. Subsequently, circular defects of 100 mm^2^ were defined at the center of each sub-region in the tibia and femur cartilage geometry, Fig 1B. Since, articular cartilage defects of at least 100 mm^2^ are considered for microfracture or mosaicplasty treatment as no spontaneous repair is expected [60]. Furthermore, previous research has indicated that important stress concentrations occur at the defect rim for defects with a diameter of 10 mm [14,61].

For each surface element, the elastic modulus and Poisson’s ratio were set. Healthy cartilage was assumed to have an elastic modulus E = 10 MPa and Poisson’s ratio ν = 0.45 [62,63]. The biomechanical changes following cartilage injury were modelled with a change in elastic modulus at the cartilage defect site whilst keeping the other parameters unchanged. An elastic modulus of 1 MPa represented softening of the cartilage layer [28,64], whereas a modulus of 100 MPa represented early stage bone ingrowth in the defect.

For each defect site and corresponding elastic modulus, the optimization problem was re-solved based on identical input data, e.g., external loads and 3D marker trajectories, to determine the contact metrics, load distribution and corresponding tibiofemoral kinematics. This resulted in a dataset containing two simulations for each defect location for each subject trial. Including the reference simulations for each subject 1110 simulations were generated in total.

To evaluate in more detail the influence of gradual changes in mechanical properties of the defect tissue on the simulated contact metrics, a sensitivity study was performed by changing the elastic modulus of the anterior-central defect in the medial femoral condyle (c1MF). In agreement with the logarithmic relationship used to calculate the pressure from the contact geometry penetration (Eqs 1 & 2), we opted for a logarithmically spaced vector (n = 27) between 1 and 100 MPa to define the elastic modulus for the sensitivity study. Softening of the cartilage was simulated with a gradual decrease in cartilage stiffness. Compaction or calcification of the cartilage matrix was simulated with increased cartilage stiffness.

### Data analysis

For each simulation, the average resultant tibiofemoral contact force, contact area and contact pressure were extracted throughout the stance phase of gait for the medial and lateral compartment separately. The contact forces were scaled to bodyweight (BW) and contact area was scaled to the dimensions of the original model (scale factor^2^). Additionally, the average compressive strain, i.e., penetration depth divided by cartilage thickness, was computed for the tibia and femur cartilages. The outcome variables contact force, contact area, contact pressure and compressive strain in medial and lateral compartment for each elastic modulus were analyzed using generalized linear mixed model design based on repeated measures with defect location as fixed factor and subjects as random factor. Because of the perturbation design of this study, each subject served as his own control. When a significant effect for defect location was found, the difference in average pressure and compressive strain inside and outside the defect of the affected compartment were further analyzed using generalized linear mixed model with repeated measures for each defect location. The models included elastic modulus as fixed factor (e.g., reference, 1MPa and 100MPa) and subjects as random factor. For one representative defect location, anterior-central medial femur defect (c1MF), differences in contact metrics between gradual changes in elastic modulus of the defect tissue and reference simulation were evaluated using a generalized linear mixed effect model. In this model, elastic moduli were included as fixed factors and subjects as random factor. The significance p-value threshold was set at 0.05 and was adjusted with Bonferroni multiple testing correction for all models. Results are expressed as mean and standard deviation. All statistical tests were conducted in MATLAB (MATLAB 2017, The MathWorks, Inc., Natick, Massachusetts, USA).

To evaluate the consistency between the reference simulation and defect conditions, kinematic waveforms of the secondary tibiofemoral degrees of freedom throughout the stance phase of gait were analyzed (reported in S1 Supporting Information). Additionally, the muscle activations were compared across simulations and no important differences were observed.

## Results

### Load bearing area

**Fig 2:**
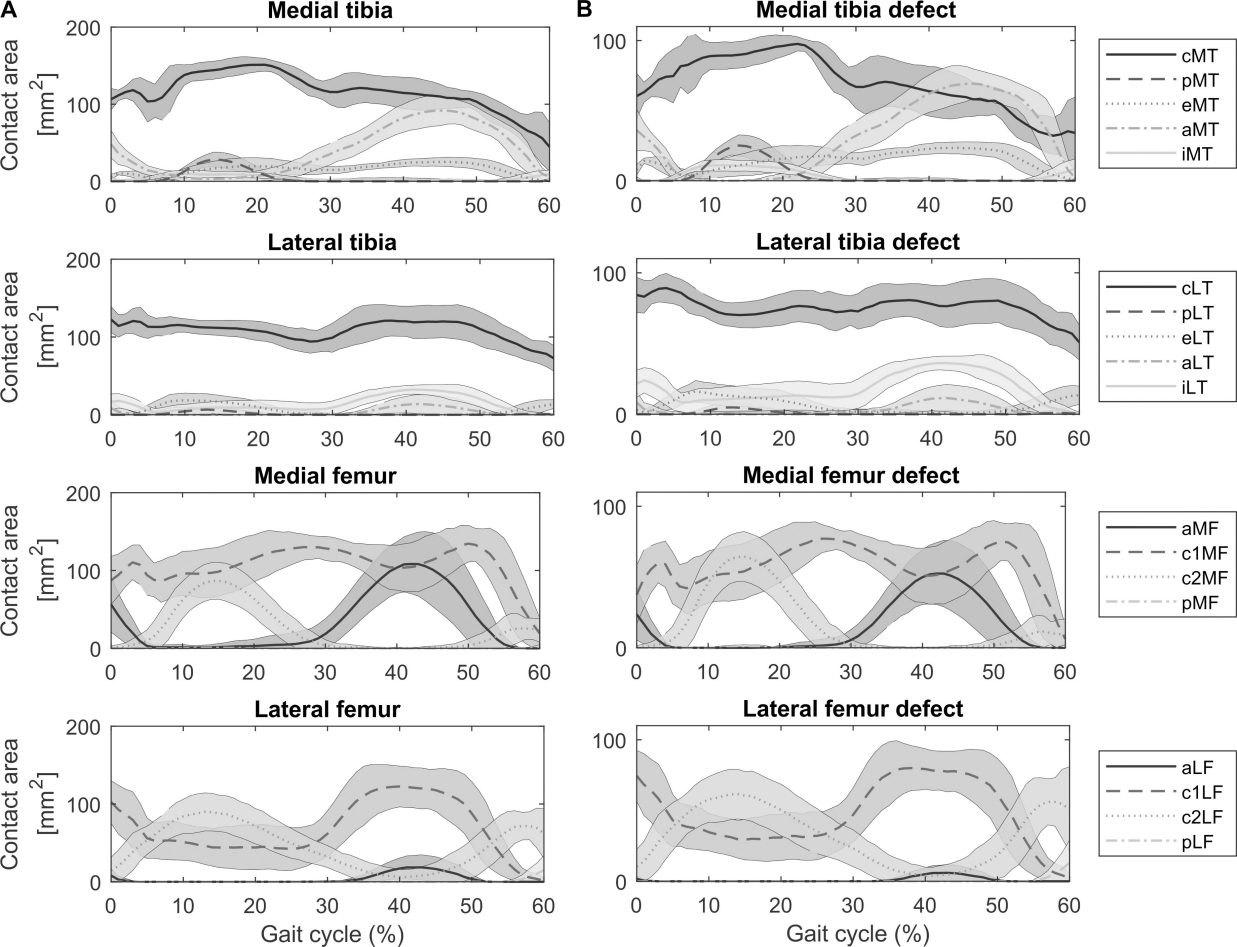
Cartilage contact area for different cartilage regions throughout the stance phase of gait. (A) Cartilage contact areas in the medial and lateral compartments of the tibia and femur for the different regions of interest throughout the stance phase of gait as defined in Fig 1. (abbreviations: MT–medial tibia, LT–lateral tibia, MF–medial femur, LF–lateral femur, c–central, p–posterior, e–external, a–anterior, i–internal, c1 –anterior-central, c2 –posterior-central). (B) Coverage of 100mm^2^ cartilage defects throughout the stance phase of gait.

Throughout the stance phase of gait, the load was mainly distributed over the central areas of the tibia (cMT & cLT) and the anterior-central regions of the femoral condyles (c1MF & c1LF). During loading response and mid stance, the posterior-central regions of the femur (c2MF & c2LF) were loaded as well, whereas in terminal stance phase the load was also transferred to the anterior medial femoral condyle (aMF) and anterior medial tibia plateau (aMT) (Fig 2A). Cartilage defects located in the aforementioned regions were therefore also partially loaded throughout the stance phase of gait. Only defects located at the central region of the tibia plateaus experienced contact over 90% of the defect area (cMT & cLT) at individual time points of the gait cycle (Fig 2B). The overall cartilage contact area of the medial compartment was larger than the lateral compartment throughout the stance phase of gait.

### Compartmental loading

**Fig 3:**
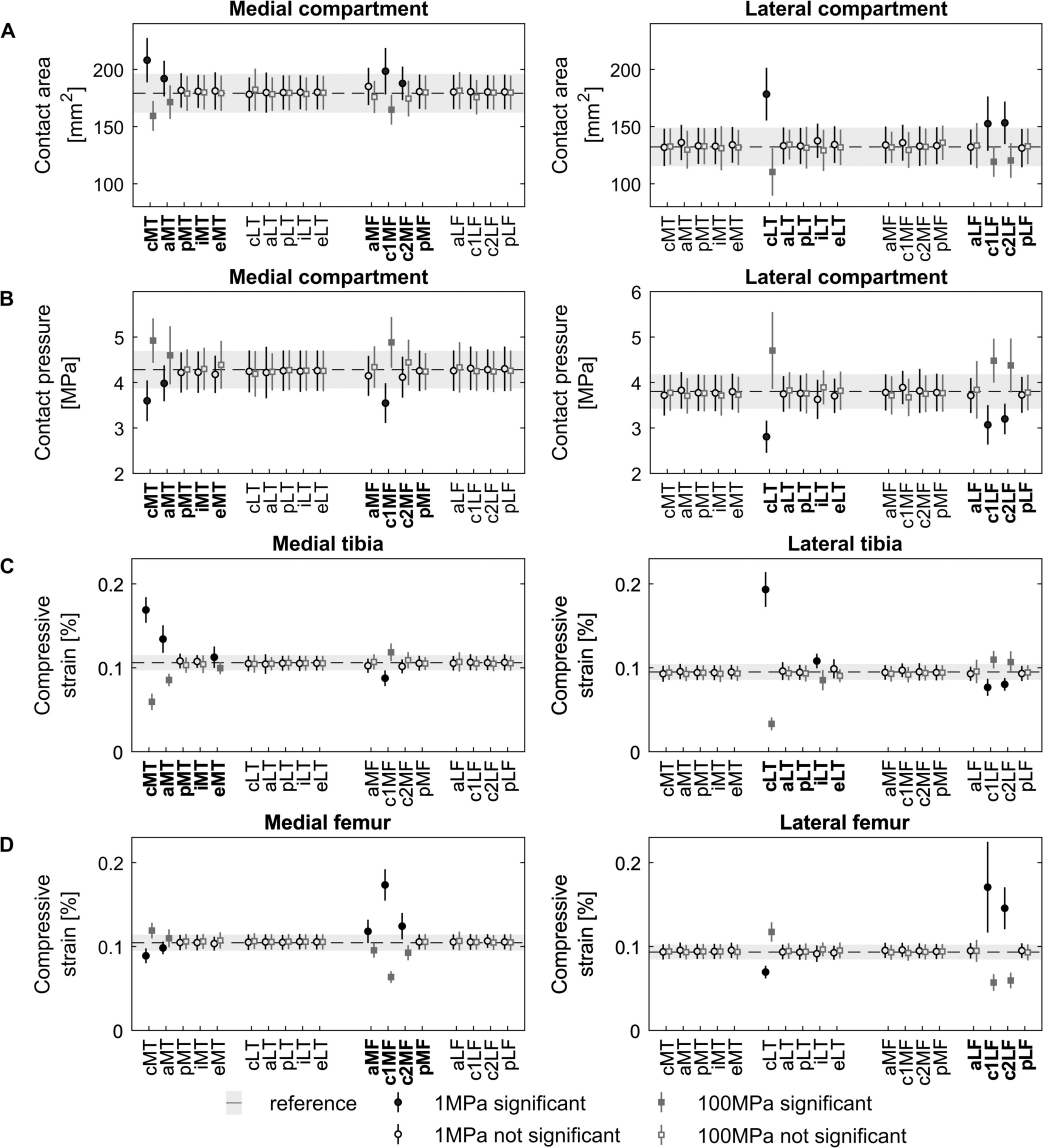
Effect of cartilage defect location and stiffness on compartmental load and compressive strain distribution. Average tibiofemoral contact area (A), pressure (B) and compressive strain in the tibia (C) and femur (D) for the different cartilage defect locations (Fig 1) throughout the stance phase of gait in the medial and lateral compartment of the tibiofemoral joint. The contact area was scaled to knee dimensions (scale factor^2^). Bold labels on the x-axis indicate defects located in the compartment used in the analysis. The dashed black line and shaded light-gray area indicate the mean and standard deviation of the reference simulations (n = 30). The black and dark-gray error bars represent the cartilage softening (1MPa) and hardening (100MPa) at the defect location, respectively. Filled marker signs indicate significant differences compared to the reference simulation (light-gray bar) at 5% significance level.

The average resultant contact force throughout the stance phase was not significantly affected by change in elastic modulus of the surface elements at any of the defect locations. Only softening of the anterior-central femoral defect in the medial compartment (c1MF) resulted in a significantly lower (p = 0.004) average resultant contact force during stance, 1.20±0.18 versus 1.30±0.15 BW (S1 Fig). Changes in elastic modulus at defect locations in the load bearing area only affected the load distribution, i.e. contact pressure and contact area, of the modified compartment (Fig 3). A decrease in elastic modulus of defects in the load bearing area resulted in an increase in contact area and consequently a lower overall contact pressure. For defects in the load bearing area presenting an increase in elastic modulus the opposite trend was observed.

The magnitude of change in contract pressures and areas was strongly influenced by the location of the defect and its relation to the load bearing area (Fig 3). Large alterations in contact metrics were observed when a large percentage of the defect area was located within the load bearing area for a longer period of the stance phase (Fig 2B and Fig 3). Tibia defects located in the central areas of the compartment (cMT & cLT) presented the largest response difference in contact area and contact pressure with changes in elastic modulus. Similar results were observed for defects located in the anterior-central regions (c1MF & c1LF) and lateral posterior-central region of the femur (c2LF). For example, a 10 times reduction in elastic modulus of the central lateral tibia defect (cLT) resulted in 34.95±7.50% (p<0.001) increase in contact area and 26.67±4.04% (p<0.001) decrease in contact pressure in the lateral compartment. However, a 10 times increase in elastic modulus of the same defect resulted in a reduction of 18.10±7.16% (p<0.001) in contact area and 25.25±13.85% (p<0.001) increase in contact pressure.

The estimated average reference strain in tibia and femoral cartilage is 10.43±0.93% and 9.34±0.86% for medial and lateral compartment respectively. An increase in elastic modulus resulted in a decreased compressive strain of the affected cartilage segment. The tissue opposing the defect presented an increase in average compressive strain, although to a lesser extent (Fig 3C and 3D defects cMT, aMT, cLT, c1MF, c1LF and c2LF). Additionally, the eMT, aMF, c2MF and iLT defects presented a significant difference in compressive strain in the affected compartment. However, this did not result in a significant difference at the opposing tissue, nor in average contact pressure (Fig 3).

### Load distribution defect and surrounding tissue

**Fig 4:**
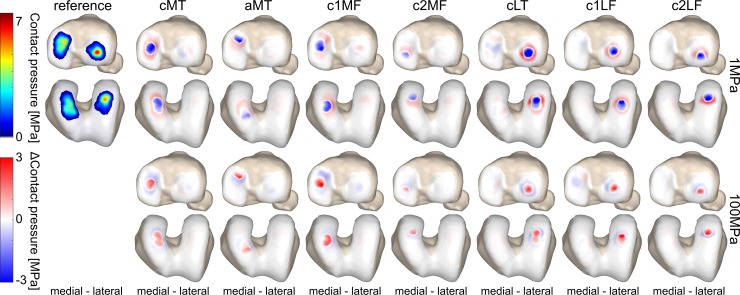
The effect of defect location on the local load distribution in the tibiofemoral joint. The average contact pressure distribution of a representative sample throughout the stance phase of gait is shown, as well as the difference pattern between the defect locations (columns) with an elastic modulus of 1MPa and 100MPa (rows) and the reference simulation. Only defect locations that presented significant differences in the average contact area and pressure at compartment level (Figs 1 & 3) are displayed. Red indicates more loading with respect to the reference simulation, blue indicates less loading with respect to the reference simulation.

**Fig 5:**
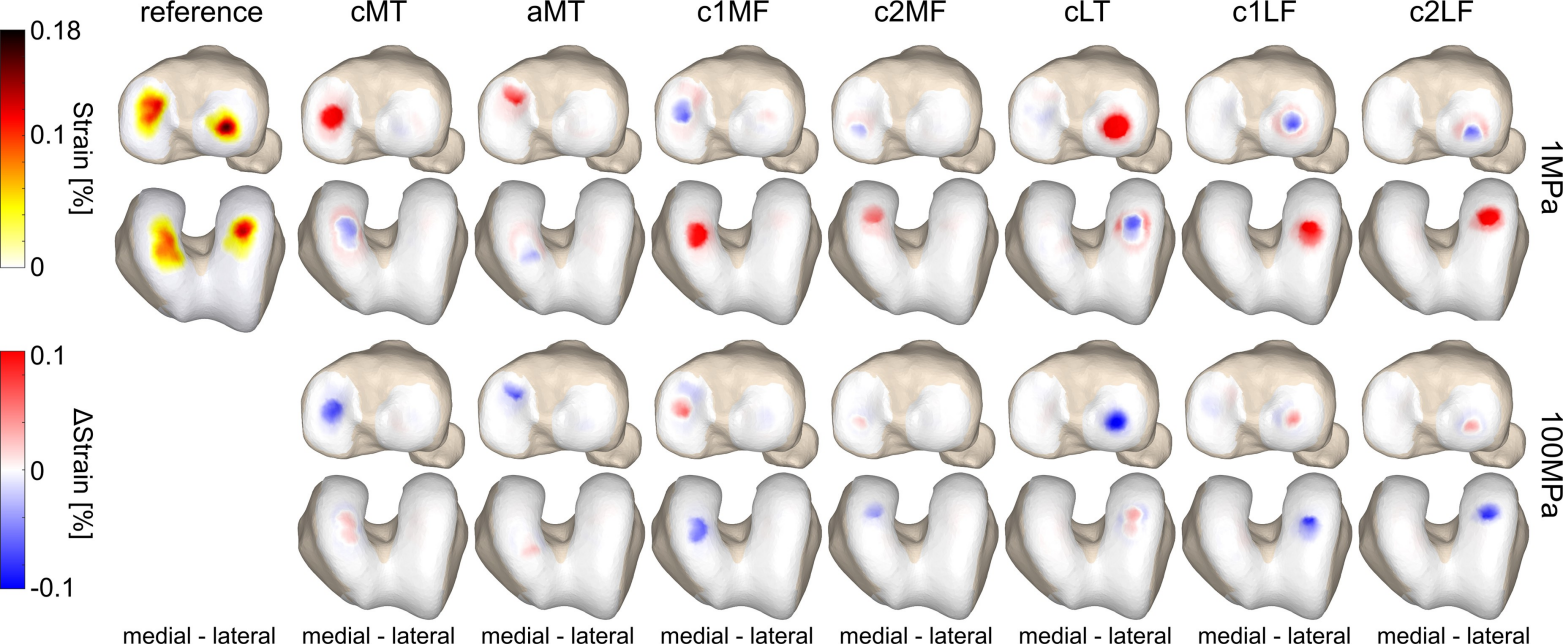
The effect of defect location on the local strain distribution in the tibiofemoral joint. The average strain distribution of a representative sample throughout the stance phase of gait is shown, as well as the difference pattern between defect locations (columns) with an elastic modulus of 1MPa and 100MPa (rows) and the reference simulation. Only defect locations that presented significant differences in the average contact area and pressure at compartment level (Fig 1 and Fig 3) are displayed. Red indicates higher strain compared to the reference simulation, blue indicates lower strain compared to the reference simulation.

**Fig 6:**
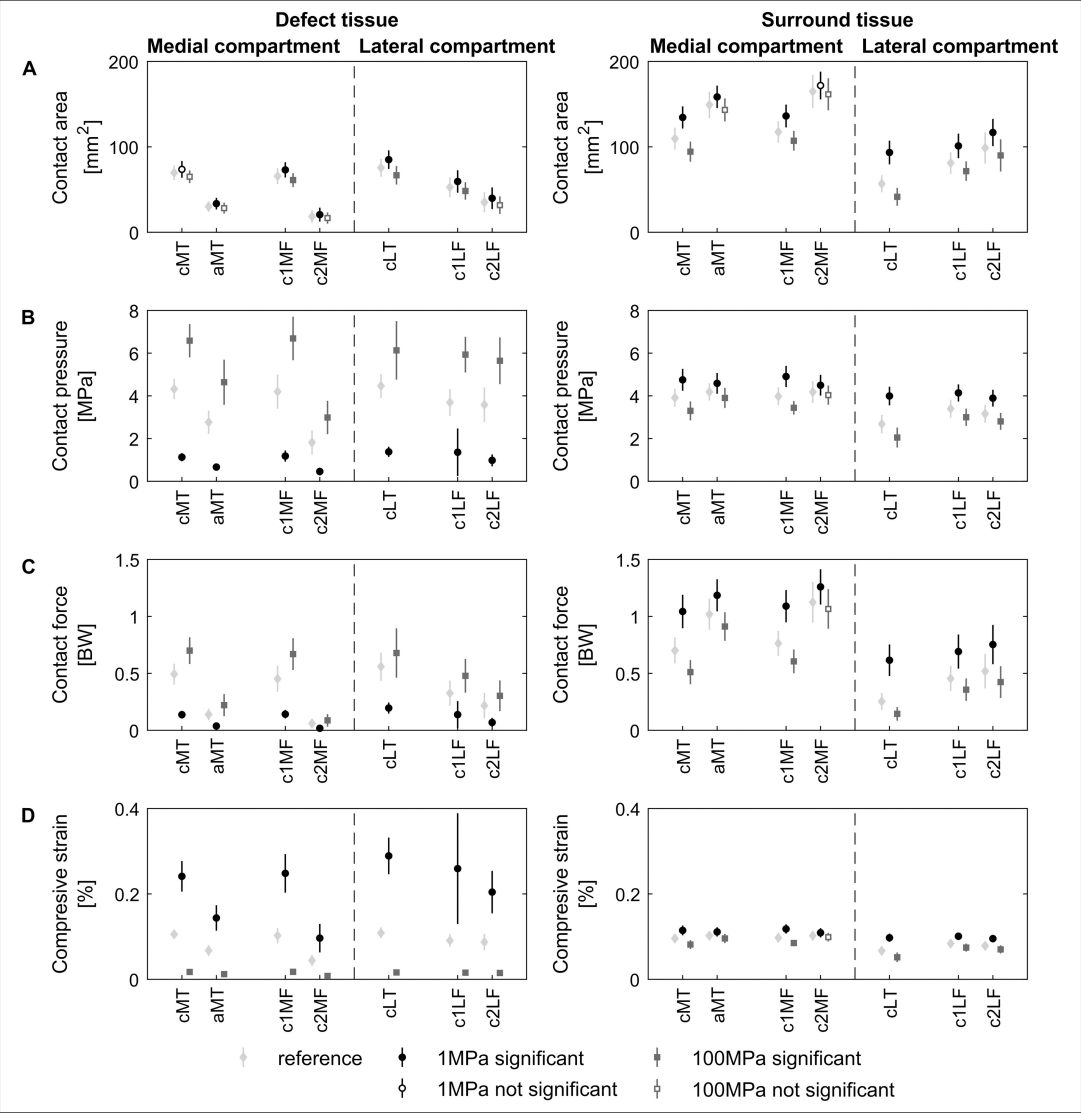
Average load distribution of defect and surrounding tissue throughout stance phase of gait. Average contact area (A), contact pressure (B), contact force (C) and compressive strain (D) inside and surrounding the defect for defect locations that presented significant differences in average contact area and pressure at compartmental level throughout the stance phase of gait (Fig 1 and Fig 3). The light-gray, black and dark-gray error bars represent the reference simulation, cartilage softening (1MPa) and hardening (100MPa) at the defect location, respectively (n = 30). Filled markers indicate significant differences compared to the reference simulation (light-gray error bar) at 5% significance level.

The influence of defect location on the local cartilage load and strain distribution in the tibiofemoral joint of a representative sample is shown in Fig 4 and Fig 5. The contact force distribution, throughout the stance phase, was more affected when a larger part of the contact area covered the defect location (Fig 6A and Fig 6C). A decrease in elastic modulus at the defect location significantly decreased the contact force on the defect tissue, whereas the contact force on the surrounding tissue significantly increased.

When evaluating the load redistribution inside and outside a defect with decreased elastic modulus, the contact pressure inside the defect significantly decreased, whereas the contact pressure of the surrounding tissue significantly increased (Fig 4 and Fig 6B). For all cases the changes in contact pressure observed inside the defect were larger compared to changes of the surrounding tissue. However, the changes in overall contact area were mainly observed outside the defect area (Fig 6A). Furthermore, the compressive strains were at least two times higher inside the defect for a tenfold decrease in elastic modulus. A tenfold increase in elastic modulus at the defect location resulted in 80% and 30% decrease of compressive strain inside and outside the defect, respectively (Fig 5 and Fig 6D). Additionally, the opposing tissue presented a decrease in compressive strain at locations that were in direct contact with the defect and an increase in strain without direct contact (Fig 5).

### Sensitivity

**Fig 7:**
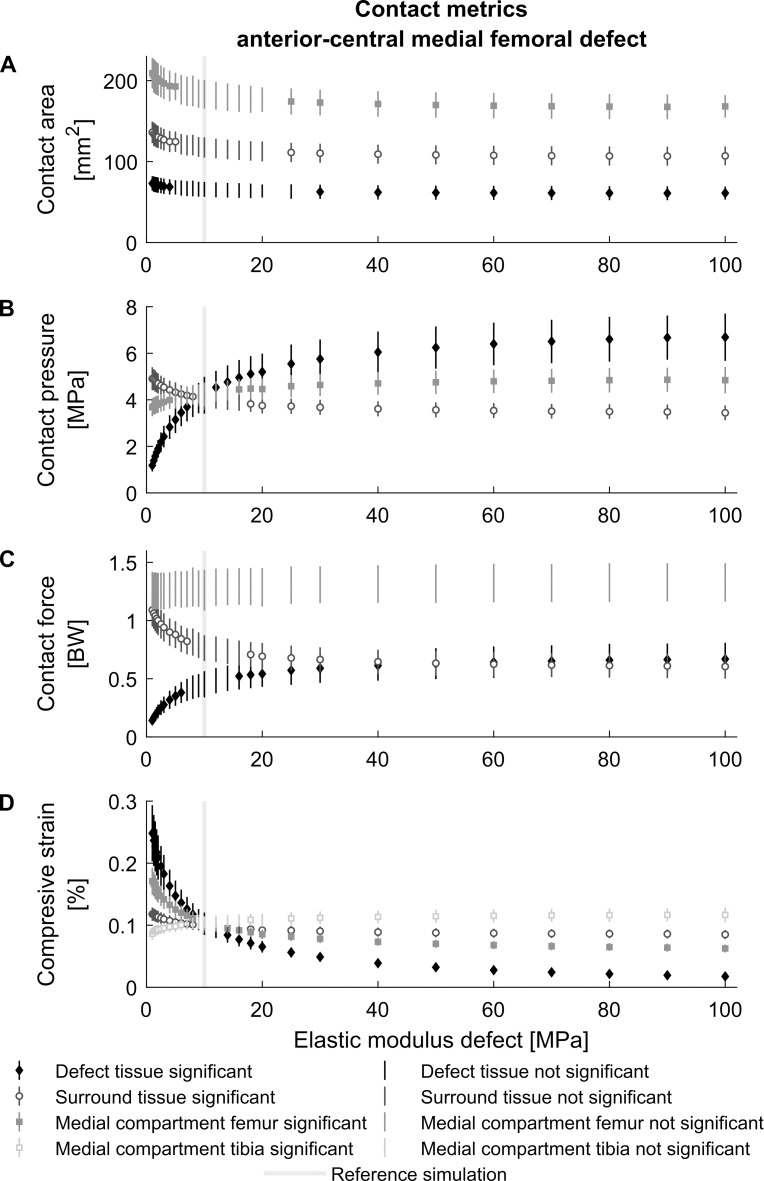
The influence of defect stiffness on local contact metrics. The effect of different elastic moduli on the average contact pressure (A), contact area (B), contact force (C) and average compresssive strain (D) inside and surrounding the defect and medial compartment of the anterior-central defect in the medial femur throughout the stance phase of gait (mean ± standard deviation). Significant differences compared to the reference simulation (gray line) at 5% significance level are indicated by dots on the error bar.

The elastic moduli of 1 MPa and 100 MPa to simulate cartilage softening and hardening resemble the extreme values of the elastic behavior within a defect. The sensitivity analysis presented a hyperbolic relation between the contact parameters and the elastic modulus at the defect location reaching asymptotic values around 1 MPa and 100 MPa (Fig 7B). Small changes in elastic modulus at the defect location have a large impact on the contact pressure and compressive strain inside the defect. Defect pressure and strain were not significantly altered for elastic moduli between 8–10 MPa and 9–12 MPa, respectively. Similar results were observed for surrounding tissue, which was more sensitive to a decrease in elastic modulus rather than an increase. The surrounding contact pressure, contact force and compressive strain were significantly altered when the elastic modulus was below 7 MPa or above 18 MPa (Fig 7B–7D). Likewise, the surrounding contact area was significantly altered for elastic moduli below 5 MPa or above 25 MPa (Fig 7A).

## Discussion

The purpose of this study was to investigate the influence of changes in local mechanical stiffness, representative for cartilage defects, on the tibiofemoral load distribution during the stance phase of gait and more specific the loading on the defect and surrounding tissue. Our results suggest that coverage of the articular cartilage defect affects the pressure and strain distribution within the involved compartment. The stiffness of the cartilage matrix at the defect location also changed the average contact area when the defect was located in the load bearing area. These changes in local mechanical properties will affect the contact mechanics and may ultimately influence the joint homeostasis by altering the mechanobiological response of the damaged, surrounding and opposing tissues and consequently initiate degenerative changes potentially evolving to OA.

Cartilage defect location and stiffness did not affect the overall joint loading (i.e., resultant contact force) under similar kinematics and kinetics (S1 Fig). Similar results in resultant contact forces were already previously observed between patients with medial and lateral tibiofemoral cartilage defects and asymptomatic controls after adjusting for walking speed [65,66]. The gait pattern of those patients was similar to asymptomatic controls [65,66]. However, our results show that defects located in the load bearing area of the tibiofemoral joint significantly affect the load distribution, specifically average contact pressure and contact area, within the involved compartment but not in the uninvolved compartment. These results are similar to Peña *et al*., 2007 [40]. As local loading is important to maintain cartilage homeostasis, the findings of this study are important to consider when using computational models to investigate the impact of cartilage damage and cartilage regenerative treatments.

Comparable to literature [67,68], a larger portion of the resultant contact force was distributed over the medial compartment during gait, resulting in higher contact pressures and larger contact area (Fig 3A and 3B). Furthermore, during the stance phase of gait the central regions of the tibia plateau and the anterior-central part of the femoral condyles were covered most compared to other regions within the tibiofemoral joint (Fig 2). As a result, defects located in the central part of the tibia plateau and anterior-central part of the medial femur experienced the greatest loading, particularly defects which presented largest defect coverage and highest contact pressures. This makes them more vulnerable to initiation of degenerative processes compared to other defect locations in lower weight-bearing areas. Indeed, previous research has indicated that the progression of defects towards OA in the medial compartment was more likely compared to lateral compartment defects when left untreated or following microfracture and ACI [13,32,33].

**Fig 8:**
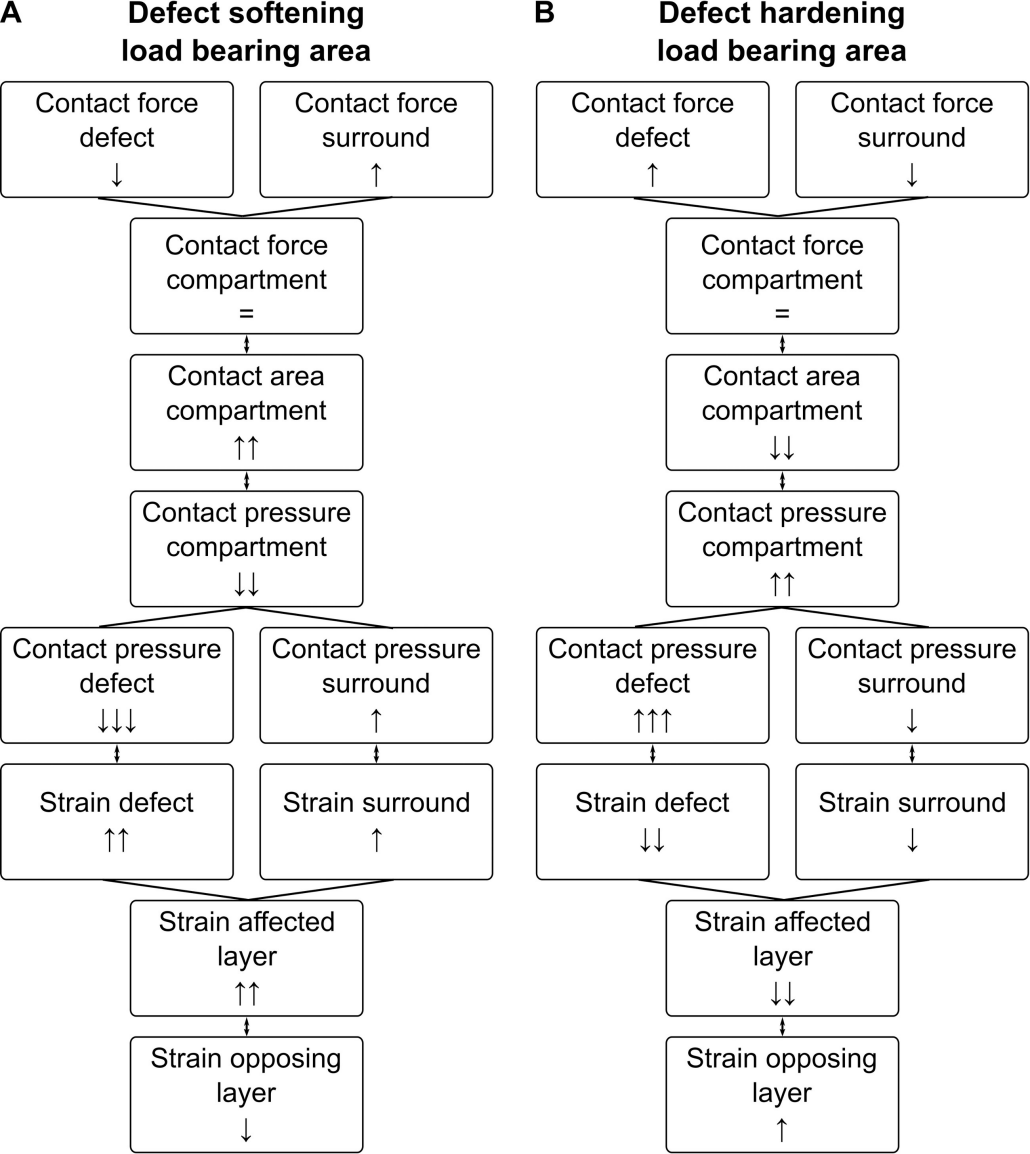
Effect of softening and hardening of a cartilage defect in the load bearing area.

Softening of the cartilage in the load bearing area significantly decreased the contact pressure within the defect location, but significantly increased the contact pressure and contact area of the surrounding tissue. Overall, the resultant contact force distribution was shifted more towards the surrounding tissue and unloaded the cartilage defect (Fig 6C). Furthermore, the involved cartilage surface presented a significant increase in compressive strain for the defect region and to a lesser extent for the surrounding tissue. Interestingly, the opposing cartilage layer presented a significant decrease in compressive strain at locations that were in direct contact with the defect tissue. The opposite loading pattern occurred when cartilage hardening was present (Fig 8B); resulting in a smaller contact area, higher contact pressure, and increased strains in the opposing tissue. Softening of the cartilage gave similar results to *in vitro* studies which observed increased pressure [14,37,38] and strains [15,69–71] around the rim of full-thickness chondral defects. Contrarily, hardening due to positioning of metal resurfacing implant resulted in increased pressure at the defect location itself [39]. These changes in load distribution can explain the progression of articular cartilage defects to OA [14–16]. Additionally, tissue opposing damaged cartilage presented signs of early degeneration in a goat model with intact joint stability, 20 weeks after defect creation [72]. Similar results were observed for cartilage tissue opposing a metallic resurfacing implant in a goat and sheep model, 1-year after surgery [73,74]. For further validation of these insights *in vivo* in subjects presenting isolated cartilage defects in the knee joint, the longitudinal use of fluoroscopy [75] and dynamic MRI [76], including DENSE imaging [77], should be considered to non-invasively estimate the local material properties based on estimated or measured deformation.

The sensitivity analysis confirms the functional role of cartilage to distribute the load over the articular surface. With an increase in mechanical stiffness, due to subchondral bone overgrowth [30] or metallic implants [78], this function is hampered as the mechanical load is transferred over a smaller contact area at the expense of an increased contact pressure. On the other hand, a decrease in mechanical stiffness, through cartilage or subchondral bone damage [26] or formation of fibrocartilage [28,29] resulted in an increase in contact area and decrease in average contact pressure (Fig 7). The sensitivity analysis indicates that local softening of the articular layer is more detrimental in altering mechanical environment, which was also observed by Shirazi & Shirazi-Adl, 2009 [43]. Especially, the increased strains within the defect and adjacent to the defect could result in the initiation and acceleration of degenerative processes at previously healthy cartilage. This might partially explain why partial thickness defects also develop to OA [21]. However, the load redistribution reached almost asymptotic values for softening and hardening (Fig 7). This is a result of the elastic foundation cartilage material model definition, which holds a logarithmic relationship between pressure and penetration depth (Eqs 1 & 2) [50,51].

Aberrant loading patterns, e.g., reduced loading and overloading, will induce changes to the anabolic and catabolic activities of chondrocytes, which change the homeostasis and can lead to either matrix remodeling or degeneration [79,80]. Research showed that reduced loading and overloading shift the homeostatic balance in chondrocytes to favor catabolic activity over anabolism resulting in cartilage degeneration. As a result, reduced loading presented thinning of the articular cartilage layer [81], whereas overloading caused direct damage to the extracellular matrix [82], chondrocyte apoptosis [83,84] and upregulation of catabolic enzymes [80,85]. Consequently, overloading will cause a direct impact on the load redistribution within the joint.

A few model limitations need to be addressed while interpreting the results of the current study. Firstly, the defects were incorporated as a change in elastic material properties of the articular surface, whilst the contact geometry and thickness were not adjusted. Consequently, the effect of volumetric loss of tissue and geometrical changes of the articular surface as clinically present with cartilage defects on the load distribution were neglected. Nonetheless, the changes in contact pressure distribution after defect creation were consistent with finite element studies incorporating more geometrical detail and different material models [41–44,78]. Our use of an elastic foundation contact model enabled the use of complex geometries whilst simulating large range of motion under physiological loading conditions and realistic movement pattern. Secondly, the model did not account for geometrical variability across subjects. Instead, a generic model including a knee joint based on one healthy female subject was scaled to the subject dimensions and mass. Keeping a consistent contact geometry allowed systematic investigation of the effect of defect location with differing input gait data. Subject-specific variations in joint congruity and geometry and their effect on the load distribution at specific locations in the knee joint, are currently omitted. Therefore, further investigation is necessary to understand the effects of subject-specific geometry. Thirdly, the knee model did not include menisci, which have a load distribution function in the tibiofemoral joint. Models including menisci showed that approximately 20% of the contact force is transmitted through the menisci during the stance phase of gait resulting in a larger contact area and causing a reduction in average contact pressure [40,86]. Therefore, potentially more defect locations located in the low-weight-bearing area covered by the menisci will be in contact and subjective to changes in contact mechanics [44]. The defects in contact with the menisci would likely show similar trends in load distribution as defects in the weight-bearing area for the affected cartilage, but minimal effect on the opposing cartilage due to the load transmission through the menisci. Lastly, the used optimization algorithm did not incorporate EMG-activations and therefore muscle co-contractions. This effect is expected to be limited as an experimental study by Thoma et al., 2016 [87] in patients with articular cartilage defects revealed no change in muscle co-contraction patterns compared to asymptomatic controls.

Despite the limitations the current workflow can be used in future research. For example, by analyzing functional activities and exercises used in the current rehabilitation programs after cartilage defects, this framework could be used to define personalized treatment strategies and rehabilitation protocols tailored on defect location for optimal restoration of the cartilage function [88,89]. A first step in this process would be the creation of a database of different movements for which the load distribution within the tibiofemoral joint is analyzed [90]. By the inclusion of different defect locations, even in a generic model, the load distribution around the defects could be analyzed, from which the medical specialist can select the most favorable to the patient, thereby avoiding overloading of the cartilage tissue in a more evidence-based manner. This would especially be suitable for patients of which the defect location is known, via either MRI or arthroscopy that is already routinely acquired in clinics as part of the clinical decision making process. Additionally, statistical shape models for personalization of the contact geometry could be included in the model to further individualize the treatment recommendation. Further personalization, e.g., localized muscle weakness, ligament laxity and contact parameters, would require the creation of subject-specific musculoskeletal models, a process that is highly time consuming.

The present study provides a comparative analysis of load distribution in the tibiofemoral joint, e.g., contact pressure, contact area, compressive strain, for cartilage defects at different locations in the tibia plateau and femoral condyles under physiological loading conditions. Local mechanical changes caused by matrix softening or hardening within the high load bearing area resulted in aberrant loading conditions, which affects the homeostatic balance within the tibiofemoral joint at not only the defect but also tissue surrounding and opposing the defect. Furthermore, small changes in local contact stiffness due to cartilage softening will alter mechanical environment within the tibiofemoral joint more compared to hardening. Nonetheless, cartilage softening and hardening predispose tissue to loads that can contribute to accelerated risk of cartilage degeneration and the initiation or progression towards OA of the whole compartment.

## Supporting information

S1 Supporting Information Effect of cartilage defect location and stiffness on knee kinematics. (DOCX)

S1 Fig Effect of cartilage defect location and stiffness on resultant compartmental contact force. Average tibiofemoral contact force for the different cartilage defect locations (Fig 1) throughout the stance phase of gait in the medial and lateral compartment of the tibiofemoral joint. The resultant contact force was scaled to body weight (BW). Bold labels on the x-axis indicate defects located in the compartment used in the analysis. The dashed black line and shaded light-gray area indicate the mean and standard deviation of the reference simulations (n = 30). The black and dark-gray error bars represent the cartilage softening (1MPa) and hardening (100MPa) at the defect location, respectively. Filled marker signs indicate significant differences compared to the reference simulation (light-gray bar) at 5% significance level.
(TIF)

S1 Table The correlation coefficients between the reference and defect kinematics throughout the stance phase. (XLSX)

S2 Table The root mean squared difference between the reference and defect kinematics throughout the stance phase. (XLSX)

S3 Table Raw data compartimental load distribution and compressive strains. Supporting data Fig 3.
(XLSX)

S4 Table Raw data load distribution of defect and surrounding tissue throughout the stance phase of gait. Supporting data Fig 6. Average contact area [mm^2^]; Contact pressure [MPa]; Force [BW]; Strain [%].
(XLSX)
