# Supplementary material for: Cartilage defect location and stiffness predispose the tibiofemoral joint to aberrant loading conditions during stance phase of gait
Source: PLoS One. 2018 Oct 16;13(10):e0205842. doi: 10.1371/journal.pone.0205842 (PMC6191138; doi:10.1371/journal.pone.0205842)
Supplement: S1 Supporting Information — (DOCX) [file pone.0205842.s001.docx]

Supplementary information kinematics

# Methods

Kinematic waveforms of the secondary tibiofemoral degrees of freedom throughout the stance phase of gait were analyzed. Differences between the reference simulation and defect conditions after COMAK were quantified by means of the Pearson correlation coefficient (R) and root mean square deviations (RMSD). R measures the shape similarity between two kinematic waveforms, whereas RMSD is a quantification of absolute difference between those waveforms.

# Results

Following changes in cartilage tissue properties, the secondary tibiofemoral kinematics presented minimal differences between the reference simulations and the defect conditions; The average RMSD throughout the stance phase was between 0.03 - 0.77 mm for the translational degrees of freedom and 0.07 – 1.01 degrees for the rotation and adduction angles. The correlation coefficients between the reference kinematics and defect kinematics throughout the stance phase of gait were always greater than 0.9. Overall, for defects in the load bearing area, the lowest correlation coefficients and largest deviations in kinematics were observed. Correlation coefficients and RMSD for each condition are presented in Table in S1 Table and S2 Table.

# Discussion

The difference in the kinematic waveforms between the reference and perturbed simulations were very small and below the previously reported measurement errors inherent to 3D gait analysis of at least 3 degrees [1]. This was expected because the kinematic and kinetic input data was similar for the reference and defect simulations. Therefore, the differences were only due to the changes in the model, more specific the changes in elastic modulus at the defect location. Nonetheless, those subtitle changes in joint kinematics and changes in contact metrics are potentially relevant when investigating the causes of early-onset osteoarthritis. Similarly, a previous sensitivity study on the influence of ligament properties on knee mechanics during the stance phase of normal walking showed that translations up to 3 mm and internal-external rotations of 5° [2] could occur.

[1] Schwartz MH, Trost JP, Wervey RA. Measurement and management of errors in quantitative gait data. Gait Posture 2004;20:196–203. doi:10.1016/j.gaitpost.2003.09.011.

[2] Smith CR, Lenhart RL, Kaiser J, Vignos MF, Thelen DG. Influence of Ligament Properties on Tibiofemoral Mechanics in Walking. J Knee Surg 2015;1:99–106. doi:10.1055/s-0035-1558858.
