# Supplementary figures and images for: Cartilage defect location and stiffness predispose the tibiofemoral joint to aberrant loading conditions during stance phase of gait
Source: PLoS One. 2018 Oct 16;13(10):e0205842. doi: 10.1371/journal.pone.0205842 (PMC6191138; doi:10.1371/journal.pone.0205842)

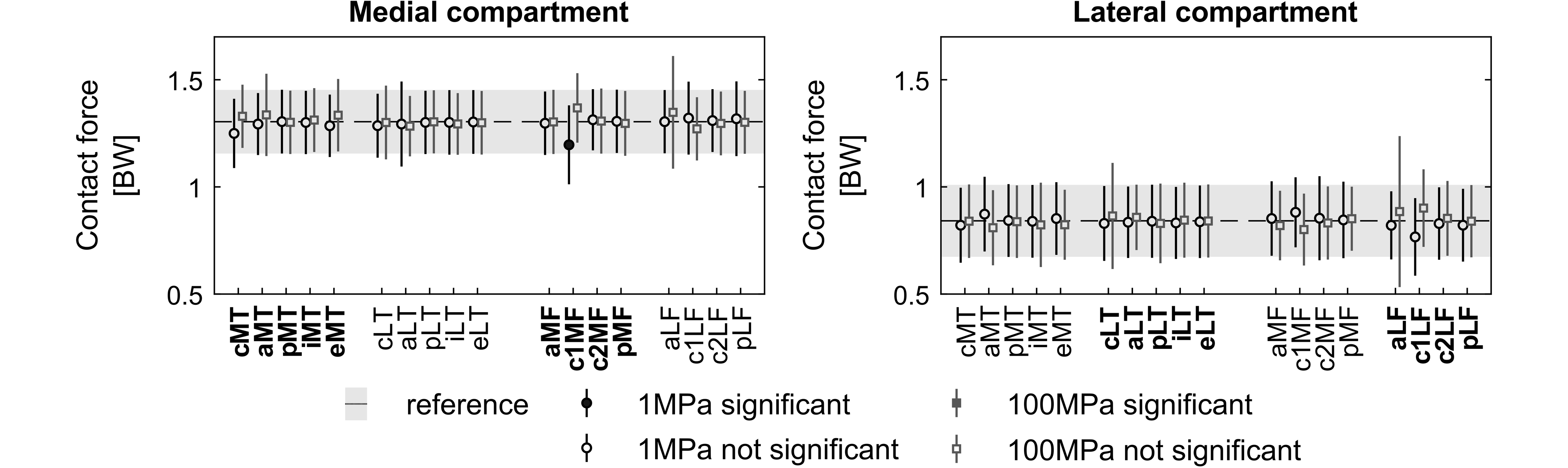

Supplement: S1 Fig — Average tibiofemoral contact force for the different cartilage defect locations (Fig 1) throughout the stance phase of gait in the medial and lateral compartment of the tibiofemoral joint. The resultant contact force was scaled to body weight (BW). Bold labels on the x-axis indicate defects located in the compartment used in the analysis. The dashed black line and shaded light-gray area indicate the mean and standard deviation of the reference simulations (n = 30). The black and dark-gray error bars represent the cartilage softening (1MPa) and hardening (100MPa) at the defect location, respectively. Filled marker signs indicate significant differences compared to the reference simulation (light-gray bar) at 5% significance level. (TIF) [file pone.0205842.s002.tif]
